# Supplementary material for: Signal and noise in metabarcoding data
Source: PLoS One. 2023 May 11;18(5):e0285674. doi: 10.1371/journal.pone.0285674 (PMC10174484; doi:10.1371/journal.pone.0285674)
Supplement: S1 File — (DOCX) [file pone.0285674.s001.docx]

**Supplement 1: A note on sampling depth and the probability of observing zeros.**

In the metabarcoding literature, it is often asserted that if more reads are sampled, it is more likely that rare variants will be observed. While this is true, the magnitude of the effect is likely less than one would like and only meaningfully changes the probability for a relatively narrow range of rare things. This supplement provides some simple examples for how to do that calculation.

Let us focus on only the last step of the metabarcoding process: multinomial sampling. As we are interested in isolating the contribution of sampling depth we can assert that everything prior to the sampling of DNA strands is identical; only the sampling depth changes. So for illustration, let’s discuss a single taxon (“A”) that comprises 0.0001% of the DNA (1 in 10,000 sequences) of the post-PCR product. We will assume that multinomial sampling is a decent approximation to the process (i.e., there are so many DNA copies floating around removing a few doesn’t materially change the probability of observing a given taxon; for those who are uncomfortable with this assumption, the below can be reframed using the hypergeometric distribution in place of the multinomial). For a single taxon, the multinomial collapses to the binomial (i.e., we can think of many taxa collapsing to two groups: taxon A and not taxon A). The probability mass function for the binomial is:

$p(k|\pi,n) = \frac{n!}{k!(n-k)!} \pi^{k}(1-\pi)^{n-k}$ (1)

where k is the number of “successes” (observations of taxon A by the sequencer) and *n* is the number of sequences read. We are interested in a single value here: what is the probability of *k=0* (i.e. taxon A was not observed) as the number of sequences examined (*n*) increases. First, simplify for the case *k=0*

$p(k=0|\pi,n) =(1-\pi)^{n}$ (2)

then plug in $\pi=0.0001$ a range of values for sampling depth(below I use 10 thousand, 100 thousand, and 1 million reads):

$p(k=0|\pi=0.0001,n=10,000) =(1-0.0001)^{10000}=0.367861$ (3)

$p(k=0|\pi=0.0001,n=100,000) =(1-0.0001)^{100000}=0.00045377$ (4)

$p(k=0|\pi=0.0001,n=1,000,000) =(1-0.0001)^{1000000}=3.7\times10^{-44}$ (5)

So for a species that is rare we go from seeing 1 or greater sequences with probability of 0.64 (1-0.36) at 10,000 reads to seeing it with almost certainty at 100,000 or more reads. Let’s do the calculation for a rarer sequence.

$p(k=0|\pi=0.000001,n=10,000) =(1-0.000001)^{10000}=0.99$ (6)

$p(k=0|\pi=0.000001,n=100,000) =(1-0.000001)^{100000}=0.90$ (7)

$p(k=0|\pi=0.000001,n=1,000,000) =(1-0.000001)^{1000000}=0.367$ (8)

So for a sequence that occurs at a rate of 1 in a million you go from observing zero 99% of the time with a sampling depth of 10,000 reads to 90% at 100 thousand reads to only 36% at 1 million reads.

Going the other direction, let’s look at something that is more common, say 1 in 1,000:

$p(k=0|\pi=0.001,n=10,000) =(1-0.001)^{10000}=0.00045377$ (9)

$p(k=0|\pi=0.001,n=100,000) =(1-0.001)^{100000}=3.7\times10^{-44}$ (10)

$p(k=0|\pi=0.001,n=1,000,000) =(1-0.001)^{1000000}=0$ (11)

Thus, you are almost certain to see at least one copy at 10,000 or greater read depths.

So what does this mean in general? Basically, you will see rarer things at higher read depths but moving from a read depth of say 10,000 to 1 million will only meaningfully change non-detection of very rare sequence variants (sequences that make up somewhere between 1 in 10,000 and 1 in 1 million copies). If you think that there are a lot of taxa that you care about are in this very rare zone, it may make sense to do more sequencing. But things that are extremely rare (occur at a frequency of less than 1 in a million) still will not be detected. Note that things can be rare after PCR because they are rare in the sample or because they are poor amplifiers, or both. One caveat to the description above is it only includes the probability of observing exactly zero. Many researchers use a higher threshold to determine presence (say *k > 10*, for example). Calculating *k > K* is not quite as easy as *p(k=0)* in that more terms are involved, but it is certainly not a hard calculation and involves summing the probability of *k=0* to *k=K,*

$p(k\geq K|\pi,n) =1-\sum_{k=0}^{k=K} {\frac{n!}{k!(n-k)!}\pi}^{k}(1-\pi)^{n-k}$ (12)
