# Supplementary material for: Signal and noise in metabarcoding data
Source: PLoS One. 2023 May 11;18(5):e0285674. doi: 10.1371/journal.pone.0285674 (PMC10174484; doi:10.1371/journal.pone.0285674)
Supplement: S2 File — We changed parameters in the simulation to understand their effects on non-detection. Specifically, we tested the effect of Npcr1 = 20 rather than Npcr1 = 35 presented in the main text. We also simulated uneven DNA concentrations across species and its effect on the probability of non-detection. (DOCX) [file pone.0285674.s002.docx]

**Supplemental 2 Alternate simulation results**

**Changing N_pcr1**

It is important to understand how changing some of the parameters in the simulation affect the probability of non-detection. In Fig. S1 we used $N_{pcr1}=20$ rather than $N_{pcr1}=35$ presented in the main text. As $N_{pcr1}$ declines, the probability of non-detects becomes more similar among species and only species with amplification efficiencies that are much lower than the average $a$ (in this case $a_{i}<0.4$) have increased non-detection probabilities (Fig. S1A).

**Figure S1.** **Non-detects Driven By Both DNA Concentration and Amplification Efficiency.**

The probability of non-detection (*p(Y=0)*) is shown for a community of 50, equally abundant taxa with the amplification efficiency distribution shown inset in each panel. This simulation uses $N_{pcr1}=20$ (see Fig. 1 for the same simulation but with $N_{pcr1}=35$). The amount of among-taxa variation in amplification efficiency varies from highly variable *(A;* γ=5) to moderate variation (*B:* γ=10) to low variation (*C:* γ=100) to effectively no variation (*D:* γ=1,000,000). Both subsampling and amplification efficiencies influence the rate of non-detection. The probability of observing no DNA in a given technical replicate is highest at low DNA concentrations (<10 copies /µL). However, non-detects are possible for species with low amplification efficiencies and very likely (*p(Y=0)* > 0.5) for amplification well below average (in this case approximately $a_{i}<0.3$ *)*.

**Simulating uneven DNA concentrations**

The base simulation presented in the main text assumes that the starting DNA concentration for each taxon is equivalent (i.e., for ten taxa, each comprises 10% of the DNA in a sample). While this assumption makes it easier to visualize the simulation results, it clearly does not represent natural communities which have skewed abundance distributions (some taxa are common while others are rare). To illustrate the consequences of a skewed abundance distribution we simulated a community of 20 taxa with 2 taxa each comprising 20% of the DNA, 8 taxa each with 5% of the DNA, and 10 taxa with 2% of the DNA. Otherwise, we followed the simulation parameters described in the main text. Figure S2 presents the patterns of non-detections for a single community of 20 taxa (Figure S2A) with large among-species variation in amplification efficiency ($\gamma=5)$ and for 20 communities of 20 taxa each overlaid on one figure (Fig. S2B). Facets show the true starting proportion within each community (proportions of 0.02, 0.05, or 0.20).

As shown in the even community simulated in the main text, for all taxa non-detection increases as DNA concentration declines and taxa with lower amplification rates show higher probability of non-detection. But there is clearly an interaction between the community proportion and amplification efficiency which affects the probability of non-detection. Specifically, for two taxa with equivalent amplification efficiencies, the more abundant taxa (community proportion of 0.20) have a much lower probability of non-detection than a relatively rare species (community proportion of 0.02; Fig. S2B). Indeed, for taxa with a community proportion of 0.02, at a constant DNA concentration, $p(Y=0|\lambda$=10) > 0.5 when $a_{i}<0.4$5. In contrast, for taxa with community proportions of 0.20, $p(Y=0|\lambda$=10) > 0.5 only occurred for one taxa in the 20 simulated communities with a very low amplificiation efficiency ($a_{i}=0.19$).

Thus both community proportion and amplification efficiency affect the probability of non-detection. In broad strokes, amplification efficiency will play a more important role in determining non-detection when taxa are rare relative to other species in a sample. The importance of amplification efficiency increases with PCR protocols that use a large number of PCR cycles. Non-detection of relatively common taxa in a community will generally be less influenced by relative amplification efficiency, but non-detection can still occur if amplification efficiency is sufficiently low.

**Figure S2.** **Non-detects Driven By Both DNA Concentration and Amplification Efficiency.**

The probability of non-detection (*p(Y=0)*) is shown for a community of 20 taxa with 4 taxa comprising 0.20 of the initial DNA, 8 with 0.05 of the DNA, and 10 species comprising 2% of the DNA across a range of initial DNA concentrations. *A:* Presents results for a single 20 taxa community with facets representing the three abundance categories. *B* shows results for 20 communities of 20 taxa each to illustrate general patterns. For all simulations we use $N_{pcr1}=35$ and a fixed amount of among-taxa variation in amplification efficiency *(*γ = 5). Clearly, relative abundance influence the rate of non-detection with relatively rare taxa (those with 0.02 having larger probabilities of non-detection than common taxa (0.2) with equivalent amplification efficiencies (colors).
