# Supplementary material for: Signal and noise in metabarcoding data
Source: PLoS One. 2023 May 11;18(5):e0285674. doi: 10.1371/journal.pone.0285674 (PMC10174484; doi:10.1371/journal.pone.0285674)
Supplement: Attachment — Submitted filename: review - Copy.pdf [file pone.0285674.s006.pdf]

Review of Gold et al. (2022)

reviewer

2022-10-19

This is a very interesting paper!

I think this paper just needs some better links to the existing literature before publication.

Comments on statistical simulation

Originally I thought simulation was unnecessary, since we could just compute $P(Y = 0)$ directly. However, it's much harder than it first appears! I only got as far as an expression for X_1 (not even closed form!). I therefore agree with your approach to simulate but could you please link your work to some of the existing literature (below) on these distributions? And search the literature for anything relating to DNA amplification with regards to such distributions.

Marginal distribution of $X_1|a, N_1, \lambda$ is Equation 43 in Neyman (1939), where $m_1 = \lambda$, $m_2 = (1 + a)^{N_1}$, $z = \lambda \exp(-m_2)$. Note $N_1 = N_{\text{pcr1}}$.

$$P(x_1|a, N_1, \lambda) = \sum_{w=0}^{\infty} P(x_1|w, a, N_1)P(w|\lambda) \tag{1}$$

$$= \exp(-\lambda)\frac{(1 + a)^{N_1 x_1}}{x_1!}\sum_{w=0}^{\infty}\frac{w^{x_1}}{w!}z^w \tag{2}$$

This is called a *Neyman type A distribution* by Dobbie and Welsh (2001) (see Equation 1 in the paper), or a *Poisson-stopped sum distribution* by Bokes et al. (2011). See also Coly et al. (2016).

In particular

$$P(x_1 = 0|a, N_1, \lambda) = \exp(-\lambda)\sum_{w=0}^{\infty}\frac{z^w}{w!} \tag{3}$$

$$= \exp(-\lambda)\exp\{\lambda \exp[-(1 + a)^{N_1}]\} \tag{4}$$

It's a weird distribution! Certainly very interesting! It's difficult to work with so I just compute w to 100 (rather than infinity).

```
library(dplyr)
library(ggplot2)

x1_dist <- function(x, lambda_w = 5, a = 0.7, Npcr1 = 10, Nw_upper = 100){
```

```
  out = sum(sapply(0:Nw_upper, function(w){dpois(x, w*((1+a)^Npcr1))*dpois(w, lambda_w)}))

  return(out)
}

x_df = data.frame(x1 = 0:2500)

x_df = x_df %>% rowwise() %>% mutate(density_x1 = x1_dist(x1, lambda_w = 5))

ggplot(x_df) + aes(x = x1, y = density_x1) + geom_line()
```

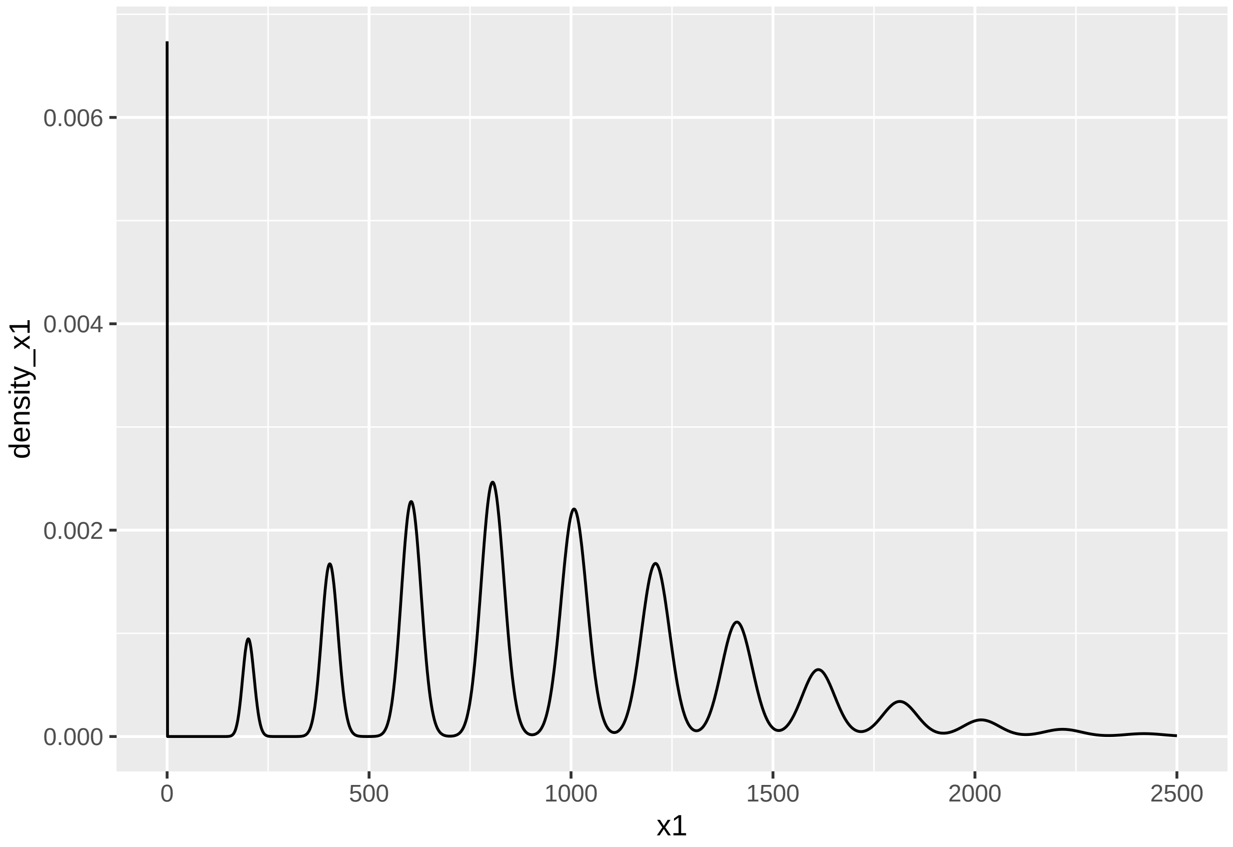

Other comments

Figure 1.

I think the inset is difficult to understand. Both axes are probabilities, but it makes more sense to label the x axis as probability. My understanding is that this is a histogram of $P(Y_i|a_i, ...)$ values, right? Maybe a plot of i vs $P(Y_i|a_i, ...)$ (ordered by so it's ascending or descending in the y axis) would be better? Or maybe a_i vs $P(Y_i|a_i, ...)$.

Explanation of approach

117. we start with a sample of extracted DNA containing λ_i DNA copies $\mu L^{-1} DNA$....

Could you write something like "Suppose there is a solution of DNA molecules of concentration λ_i copies per $\mu L\ldots$", I think this would be clearer.

Please make the functions and code in the repository easier to understand and use

Bibliography

Bokes, Pavol, John R. King, Andrew T. A. Wood, and Matthew Loose. 2011. "Exact and Approximate Distributions of Protein and mRNA Levels in the Low-Copy Regime of Gene Expression." *Journal of Mathematical Biology* 64 (5): 829–54. https://doi.org/10.1007/s00285-011-0433-5.

Coly, Sylvain, Anne-Franoise Yao, David Abrial, and Myriam Charras-Garrido. 2016. "Distributions to Model Overdispersed Count Data." *Journal de La Société Française de Statistique* 157 (2): 39–63. http://www.numdam.org/item/JSFS_2016___157_2_39_0/.

Dobbie, Melissa J, and Alan H Welsh. 2001. "Models for Zero-Inflated Count Data Using the Neyman Type a Distribution." *Statistical Modelling* 1 (1): 65–80. https://doi.org/10.1177/1471082x0100100106.

Neyman, J. 1939. "On a New Class of "Contagious" Distributions, Applicable in Entomology and Bacteriology." *The Annals of Mathematical Statistics* 10 (1): 35–57. https://doi.org/10.1214/aoms/1177732245.
