# Supplementary material for: Signal and noise in metabarcoding data
Source: PLoS One. 2023 May 11;18(5):e0285674. doi: 10.1371/journal.pone.0285674 (PMC10174484; doi:10.1371/journal.pone.0285674)
Supplement: Attachment — Submitted filename: response_to_reviewer_20221219.docx [file pone.0285674.s007.docx]

1. Please ensure that your manuscript meets PLOS ONE's style requirements, including those for file naming. The PLOS ONE style templates can be found at
   <https://journals.plos.org/plosone/s/file?id=wjVg/PLOSOne_formatting_sample_main_body.pdf> and 
   <https://journals.plos.org/plosone/s/file?id=ba62/PLOSOne_formatting_sample_title_authors_affiliations.pdf>
2. In your Methods section, please provide additional information regarding the permits you obtained for the work. Please ensure you have included the full name of the authority that approved the field site access and, if no permits were required, a brief statement explaining why.

**Line 247:** **No permits were needed for this work as we are utilizing previously published data sets.**

3. We note that the grant information you provided in the ‘Funding Information’ and ‘Financial Disclosure’ sections do not match.

When you resubmit, please ensure that you provide the correct grant numbers for the awards you received for your study in the ‘Funding Information’ section.

4. Thank you for stating in your Funding Statement: 
"ZG and AJJ were supported by the Joint Institute for the Study of the Atmosphere and Ocean (JISAO) under NOAA Cooperative Agreement NA15OAR4320063. RPK, EAA, and ED were supported by the Packard Foundation Grant (XX12345)."

Please provide an amended statement that declares *all* the funding or sources of support (whether external or internal to your organization) received during this study, as detailed online in our guide for authors at <http://journals.plos.org/plosone/s/submit-now>.  Please also include the statement “There was no additional external funding received for this study.” in your updated Funding Statement. 
Please include your amended Funding Statement within your cover letter. We will change the online submission form on your behalf.

5. Thank you for stating the following financial disclosure: 
"ZG and AJJ were supported by the Joint Institute for the Study of the Atmosphere and Ocean (JISAO) under NOAA Cooperative Agreement NA15OAR4320063. RPK, EAA, and ED were supported by the Packard Foundation Grant (XX12345)."

Please state what role the funders took in the study. If the funders had no role, please state: ""The funders had no role in study design, data collection and analysis, decision to publish, or preparation of the manuscript."" 
If this statement is not correct you must amend it as needed. 
Please include this amended Role of Funder statement in your cover letter; we will change the online submission form on your behalf.

**Line 633: We have amended our Funding Information section to match the Financial Discolure section: “ZG was supported by the Joint Institute for the Study of the Atmosphere and Ocean (JISAO) under NOAA Cooperative Agreement NA15OAR4320063. AJJ was supported by NOAA and University of Washington. RPK was supported by the Packard Foundation. EAA and ED were supported by OceanKind.** **The funders had no role in study design, data collection and analysis, decision to publish, or preparation of the manuscript.”**

6. We noted in your submission details that a portion of your manuscript may have been presented or published elsewhere. [This manuscript has been posted as a pre-print on biorxiv: <https://www.biorxiv.org/content/10.1101/2022.09.02.506420v1>] Please clarify whether this [conference proceeding or publication] was peer-reviewed and formally published. If this work was previously peer-reviewed and published, in the cover letter please provide the reason that this work does not constitute dual publication and should be included in the current manuscript.

**This work was not previously peer-reviewed and formally published. We had initially submitted the journal to PLOS Biology and had submitted it as a pre-print. However, we were referred to then submit to PLOS One and thus the generation of the pre-print prior to submission.**

7. We note that you have stated that you will provide repository information for your data at acceptance. Should your manuscript be accepted for publication, we will hold it until you provide the relevant accession numbers or DOIs necessary to access your data. If you wish to make changes to your Data Availability statement, please describe these changes in your cover letter and we will update your Data Availability statement to reflect the information you provide.

**We will provide links and DOIs upon acceptance and make sure to edit the Data Availability statement appropriately.**

[Note: HTML markup is below. Please do not edit.]

Reviewers' comments:

Reviewer's Responses to Questions

**Comments to the Author**

1. Is the manuscript technically sound, and do the data support the conclusions?

The manuscript must describe a technically sound piece of scientific research with data that supports the conclusions. Experiments must have been conducted rigorously, with appropriate controls, replication, and sample sizes. The conclusions must be drawn appropriately based on the data presented.

Reviewer #1: Yes

Reviewer #2: Yes

2. Has the statistical analysis been performed appropriately and rigorously?

Reviewer #1: Yes

Reviewer #2: Yes

3. Have the authors made all data underlying the findings in their manuscript fully available?

The [PLOS Data policy](http://www.plosone.org/static/policies.action#sharing) requires authors to make all data underlying the findings described in their manuscript fully available without restriction, with rare exception (please refer to the Data Availability Statement in the manuscript PDF file). The data should be provided as part of the manuscript or its supporting information, or deposited to a public repository. For example, in addition to summary statistics, the data points behind means, medians and variance measures should be available. If there are restrictions on publicly sharing data—e.g. participant privacy or use of data from a third party—those must be specified.

Reviewer #1: Yes

Reviewer #2: Yes

4. Is the manuscript presented in an intelligible fashion and written in standard English?

PLOS ONE does not copyedit accepted manuscripts, so the language in submitted articles must be clear, correct, and unambiguous. Any typographical or grammatical errors should be corrected at revision, so please note any specific errors here.

Reviewer #1: Yes

Reviewer #2: Yes

5. Review Comments to the Author

Please use the space provided to explain your answers to the questions above. You may also include additional comments for the author, including concerns about dual publication, research ethics, or publication ethics. (Please upload your review as an attachment if it exceeds 20,000 characters)

Reviewer #1: This is a very interesting paper!

I think this paper just needs some better links to the existing literature before publication.

\# Comments on statistical simulation

Originally I thought simulation was unnecessary, since we could just compute $P(Y=0)$ directly. However, it's much harder than it first appears! I only got as far as an expression for $X_1$ (not even closed form!). I therefore agree with your approach to simulate but could you please link your work to some of the existing literature (below) on these distributions? And search the literature for anything relating to DNA amplification with regards to such distributions.

**We thank the reviewer for their insightful comments and have made a better effort to link our work to the statistical literature. See Lines 195-200.**

See attachment for the rest of the review.

Review 1 Figure 1 Concerns with insert axes

**The x axes labels of the inserts of Figure 2 (formally Figure 1) are amplification efficiency (ai) values while the x axis is the probability. Each insert is a histogram and density plot of the amplification efficiency values used in each of the four subplots. We have amended the caption to better reflect this. Line: 383**

Reviewer #2: Overall comments: The authors in this manuscript conduct a metabarcoding analysis of both mock communities and collected DNA samples to explore the factors that impact negatives in technical replicates. It was found that starting concentration and species-specific amplification efficiency. Overall, the methods are to be sound, and the presentation of the results is well thought out (the figures are also nicely done). However, I think the manuscript needs to be adjusted to better tell the story and highlight the relationships to the other papers that are commonly cited throughout. As it is currently written it comes across as though the results are not novel but just a repetition of previous work (see comments below for specifics).

**We thank the reviewer for their feedback and have made a concerted effort to identify how this paper is distinct from the two related works. Lines 128-168 and again 240:** “**We reiterate that this manuscript builds off the work of Shelton et al. [35] and Gold et al. [40] to use the above expanded mechanistic framework to explore the effects of both subsampling and PCR processes on the patterns of non-detections. Shelton et al. [35] defined the underlying mechanistic framework for metabarcoding, demonstrating the importance of accounting for amplification efficiencies in general as well as presenting a series of case studies using mock communities to validate the underlying mechanistic framework. Gold et al. [40] demonstrated that quantitative abundance estimates can also be estimated using a joint model of both metabarcoding sequences and morphological counts of ichthyoplankton using independent abundance estimates to constrain possible amplification efficiency parameters. Both works highlight the value of accounting for amplification biases in deriving quantitative abundance estimates, but neither of these works model or account for the subsampling of rare DNA molecules from the DNA extraction prior to PCR amplification in addition to the PCR and sequencing processes. Here, we build upon these works by directly modeling the subsampling process prior to PCR amplification, directly testing the effects of subsampling and PCR processes on the patterns of non-detections.”**

Line 46: I think this paragraph could be flushed out a little more and include some more information to help set up the study and metabarcoding in general. With PLoS having such a broad audience it could be useful to just add a few sentences describing some specifics for how metabarcoding is rapidly advancing health, ecology, and conservation science. Possibly include a sentence on how metabarcoding results are more and more often influencing policy decisions, which would transition well into needing reliable estimates mentioned in the next paragraph.

**We thank the reviewer for their advice and have expanded this paragraph accordingly. See Lines 62-71.**

Line 56-56: I think that this is area that could be a bit more specific on examples instead of just listing citations. Admittedly this is more of a stylistic/writing style preference and the information itself is sound, I just think it would make it a little more gripping to read.

**Given the length of the manuscript as written we decided to not flush out any examples here. We recognize the importance of previous correlational analyses and cite them heavily throughout. Line 81.**

Line 66: This could be a great spot for a summary figure highlighting the compounding processes across the DNA collection and analysis pipeline. Including the aspects you do not focus on such as the ones mentioned in the discussion or bioinformatic or sequencing platforms (see comment below).

**We agree with the reviewer and have included a new Figure 1 to highlight the overarching compounding processes that impact observed sequencing reads from metabarcoding (Line 89).**

Line 127: Does this species-specific amplification efficiency that influences the amplicons expand to genus and family level biases that can occur simultaneously in the process? For instance, if a specific species has species-specific biases but is also part of a genus that is historically biased would that be included?

**Shelton et al. [35] explored phylogenetic signals of amplification efficiencies and found no strong correlations. In order words, species within the same genus had a wide array of amplification efficiencies despite having overall more similar sequences. We recognize that little is known about how amplification efficiencies arise and highlight this within the Discussion.**

Line 221: Would GBLOCK sequences have been more useful here to ensure no nDNA or other sources got through your nested PCR strategy? Obviously you cannot go back and change anything but I am curious if this would have been more effective or not.

**We believe that GBLOCK sequences could have been used in place of fish tissue to avoid the quantification of nDNA. We did not have funding to purchase the 58 GBLOCK sequences needed for this study.**

Line 238: Why did you have to resequence, I’m assuming the run failed due to a technical failure as is so common, but was it something that could be affecting the other runs as well to a lesser degree. I am doubtful, but it may be best to be clear why the resequencing took place.

**We clarified this section to explain the additional sequencing efforts: Line 320 “The two additional mock communities included in this study were derived from the same “coastal even” mock community pool but diluted serially by a 1:10 dilution down to 10^-4^ and 10^-5^ original concentration to explore the subsampling and PCR process effects in higher DNA concentrations.” There was no failure in the sequencing run. We wanted to explore whether we observed non-detections at very high concentrations of input DNA that were not included in the initial sequencing run.**

Line 261: These hypotheses could be expanded on a bit or shifted to be a little more novel. The idea that fewer non-detections for more abundant DNA molecules seems a little obvious the way it is currently written. I agree it’s an important question to get at, but it should be reworded a bit. If this cannot be reworded, I would recommend adding a bit more text to the intro to highlight why this may not be as obvious as one would expect. This comment also relates to my comments down below about framing the story of this manuscript around the previous work.

**We agree with the reviewer that the effect of rare molecule subsampling on non-detection is not novel on it’s own. However, no previous work has looked at the interactive effects of both amplification efficiencies and DNA concentration on non-detections in metabarcoding data. We updated our hypotheses to better address the main story we are weaving Line 359: “We explore the effects of both subsampling and PCR processes on non-detections and sequence patterns. We hypothesize that non-detections can arise from both processes, but that the dominant process depends on the rarity of the molecule. For rare DNA molecules, subsampling will drive the occurrence of non-detections, while for abundant molecules, poor amplification efficiencies during PCR will drive the occurrence of non-detections. We test these hypotheses using simulation and the mock community and CalCOFI empirical examples described above.”**

Line 303 and 197: I think having read this results section there needs to be a bit more information on these samples, what they were, did they have multiple species within them etc. I know it is also in Gold et al. but it would be helpful to repeat some of this information here. Additionally, it is a little confusing teasing out these 84 ethanol-preserved samples and the formalin preserved larvae? Same as above, where more information about the previous work in other manuscripts would help make this clearer.

**We clarified the methods section to better explain the source and composition of the samples used in this study: Line 273:”** **We note that bongo nets have two paired 505 µm nets that are concurrently sampling immediately adjacent water columns. Once a sampling tow concluded, all zooplankton contents present on one side of the bongo net were preserved in Tris-buffered 95% ethanol for metabarcoding-derived species identification and sequencing. Contents on the other side of the paired net were preserved in sodium borate-buffered 2% formaldehyde for microscopy-derived species identification and abundance (number of larvae per species per jar). This dataset yields paired samples for both metabarcoding analysis and absolute abundance counts from the same sampling event.” We also included additional information on the previous studies (see above).**

Line 303: Something that I would be interested to hear the authors point of view on is whether or not these samples actually constitute eDNA. Typically, eDNA is referred to as bulk non-targeted samples but someone could argue that larvae within an ethanol bottle would not be considered eDNA sampling. This is a topic that can be rather contentious based on the audience (similar debates take place over fecal sampling), so I am curious to hear the author’s take. It is also possible (related to my above comment) that I am not clear on what these samples actually are.

**We argue that this sampling approach is eDNA because we are not directly sampling any tissue. We pipetted the ethanol that was sitting above the mass of zooplankton within each archived samples. We recognize that there are some researchers who have very strict definitions of eDNA that do not include our indirect sampling techniques because the ethanol preservative is not “the environment”. However, we argue that this indirect approach of obtaining DNA qualifies it as such. Line 289:** “**DNA sequences were generated from the liquid ethanol pipetted off the top of 84 collection lots preserved in 90 samples as described in Gold et al. [40]. Briefly, up to 125 mL of ethanol (mean = 121 mL, n= 6 < 125 mL, min=34 mL) was filtered onto 0.2 µm PVDF filters and were extracted using a Qiagen DNeasy Blood and Tissue kit.”**

Line 394: I think this illustrates my comment above about your first hypothesis seemingly already being well established across the literature.

**We have clarified our writing throughout to highlight that this finding was not novel.**

Line 467: In addition to processes from the start of eDNA collection, it could be useful to mention the impacts bioinformatic pipeline choice and sequencer selection can have on the results as well.

**We included the following to highlight the role of bioinformatics Line 577: ”** **Furthermore, limitations in bioinformatics approaches can also compound these issues, particularly in determining which sequences are retained and analyzed [57,85–88].”**

Line 507: I apologize if I missed this, but could there be a little bit of discussion about the limitations that may arise within your analysis from only having 2 species with lower amplification efficiency. This is a fairly small sample size, especially compared to the mock community that had 15 species with lower efficiency.

**We clarified the discussion section to highlight the limitations in the CalCOFI empirical example. With regards to amplicon read counts being a function of amplification efficiency we included the following on Line 513: “This was best exemplified in the mock community dataset where species with higher amplification efficiencies had over an order of magnitude more sequence reads for a given concentration of DNA than species with lower amplification efficiencies. Although only two of the 17 species in the CalCOFI data had low amplification efficiencies, the fact that we also observed an order of magnitude difference in average sequence reads per larvae between species with higher and lower amplification efficiencies strongly supports our conclusion that amplicon reads counts are a function of amplification efficiencies in addition to starting concentration.”**

**Furthermore, we included the following line to highlight that we could not directly test the effects of low amplification efficiency on non-detections in the CalCOFI example. Line 505: “Unfortunately, we could not test these patterns in the empirical CalCOFI results because we only observed low amplification efficiencies in two species and neither of these species were present in high abundance in any sampled jars (Figure 4).”**

Line 514: I apologize in advance for the length of this comment but overall, it is not completely clear the relationship and story that is being told across the various manuscripts, and as a result, it makes this work seem less novel comparatively. To my understanding….Gold (31) is where the initial samples came from, and you are reusing the samples. It also seems like these results are from the same analysis from Gold 31(?). While the Shelton et al. (27) paper seems to have initially designed the model and tested it with mock communities. Now this current paper, is using the samples from Gold 31 and testing the models described with Shelton. Though it looks like the Shelton paper has already done some mock community testing which is repeated in this reviewed paper? Assuming my assessment is correct, this took a good bit of searching and reasoning to put together, which is not ideal for a typical reader. I know you mention occasionally in the text that certain aspects come from Shelton or Gold, but the relationship is still not made very clear. For example, the ending sentence says that “together with Shelton et al.” but again that relationship across these manuscripts is unclear and as readers we didn’t know these results were being combined with Shelton from the beginning. I do believe the information presented here is novel, but the story in the introduction needs to be told in a way that highlights the path this data is taking. For example, explicitly stating that this work is a direct continuation of Shelton et al.’s model and telling the story in the introduction to guide the reader who may not know these works. Also, its mentioned that the mock communities are from Shelton et al. did they also calculate the efficiencies for these same species or was that new to this manuscript as it is not clear how it is written? If these were already calculated in Shelton et al. it may not need to be repeated to such degree here.

**We thank the reviewer for this comment and have edited the Introduction, Methods, and Discussion section to better clarify the distinction between this manuscript and that of Shelton et al. 2022 and Gold et al. 2022. In short, the novelty of this paper is the roles of subsampling and PCR process on non-detections in metabarcoding data sets. Shelton et al. 2022 presented our model for the PCR process and demonstrated how amplification efficiencies govern the observed amplicon reads. It did not investigate or incorporate any modeling efforts of the subsampling process prior to PCR. Shelton et al. 2022 also did not explore the relationship between non-detections and amplification efficiencies. Likewise, Gold et al. 2022 demonstrated that the same underlying PCR model can be used to estimate abundances by developing a joint model between morphological larval counts and observed amplicon reads from metabarcoding. However, this work did not incorporate independent estimates of amplification efficiencies (e.g. mock communities) and instead used the amplicon and morphological data sets to jointly estimate amplification efficiencies by constraining the parameter space of the model. Here we use the underlying data sets presented in Shelton et al. 2022 (mock communities) and Gold et al. 2022 (CalCOFI empirical data set) to explore the relationship of DNA concentration and amplification efficiency on the patterns of observed read counts with a particular focus on non-detections. Please see the comment above for relevant changes made in the manuscript.**

6. PLOS authors have the option to publish the peer review history of their article ([what does this mean?](https://journals.plos.org/plosone/s/editorial-and-peer-review-process#loc-peer-review-history)). If published, this will include your full peer review and any attached files.


If you choose “no”, your identity will remain anonymous but your review may still be made public.


**Do you want your identity to be public for this peer review?** For information about this choice, including consent withdrawal, please see our [Privacy Policy](https://www.plos.org/privacy-policy).

Reviewer #1: No

Reviewer #2: No
